# HIV Exposure and Neonatal Sepsis: A Descriptive Etiological Study

**DOI:** 10.1093/ofid/ofae642

**Published:** 2025-03-10

**Authors:** Patience Atuhaire, Mary Kyohere, Valerie Tusubira, Hannah G Davies, Philippa Musoke, Musa Sekikubo, Amusa Wamawobe, Joseph Peacock, Kirsty Le Doare, PROGRESS Study Author Group, Abdelmajid Djennad, Agnes Nyamaizi, Agnes Ssali, Alexander Amone, Amusa Wamawobe, Annettee Nakimuli, Caitlin Farley, Carol Nanyunja, Christine Najuka, Cleophas Komugisha, Dan R Shelley, Edward A R Portal, Ellie Duckworth, Emilie Karafillakis, Geraldine O’Hara, Godfrey Matovu, Hannah G Davies, Janet Seeley, Joseph Peacock, Juliet Nsimire Sendagala, Katie Cowie, Kirsty Le Doare, Konstantinos Karampatsas, Lauren Hookham, Madeleine Cochet, Margaret Sewegaba, Mary Kyohere, Maxensia Owor, Melanie Etti, Merryn Voysey, Moses Musooko, Musa Sekikubo, Owen B Spiller, Patience Atuhaire, Paul T Heath, Philippa Musoke, Phiona Nalubega, Pooja Ravji, Richard Katungye, Ritah Namugumya, Rosalin Parks, Rose Azuba, Sam Kipyeko, Simon Beach, Stephen Bentley, Tim Old, Tobius Mutabazi, Valerie Tusubira, Vicki Chalker

**Affiliations:** Makerere University—Johns Hopkins University (MUJHU) Research Collaboration, Kampala, Uganda; Makerere University—Johns Hopkins University (MUJHU) Research Collaboration, Kampala, Uganda; Institute for Infection and Immunity, St. George's, University of London, London, UK; Makerere University—Johns Hopkins University (MUJHU) Research Collaboration, Kampala, Uganda; Institute for Infection and Immunity, St. George's, University of London, London, UK; Clinical Research Unit, Faculty of Infectious and Tropical Diseases, London School of Hygiene & Tropical Medicine, London, UK; Makerere University—Johns Hopkins University (MUJHU) Research Collaboration, Kampala, Uganda; Medical Research Council/Uganda Virus Research Institute and London School of Hygiene & Tropical Medicine Uganda Research Unit, Entebbe, Uganda; Department of Medical Microbiology, Makerere University, Kampala, Uganda; Institute for Infection and Immunity, St. George's, University of London, London, UK; Makerere University—Johns Hopkins University (MUJHU) Research Collaboration, Kampala, Uganda; Institute for Infection and Immunity, St. George's, University of London, London, UK; Medical Research Council/Uganda Virus Research Institute and London School of Hygiene & Tropical Medicine Uganda Research Unit, Entebbe, Uganda

**Keywords:** antimicrobial resistance, neonates, HIV exposure, neonatal infection, sepsis

## Abstract

**Background:**

Low- and middle-income countries lack data on culture-confirmed sepsis in HIV-exposed infants, despite the reported heightened risk of infectious morbidity. This study describes culture-confirmed sepsis and antibiotic resistance patterns among HIV-exposed children in a large etiological cohort study in Kampala, Uganda.

**Methods:**

This was a prospective birth cohort study based at 2 Ugandan sites, as part of the Progressing Group B Streptococcal Vaccines (PROGRESS) study. Any infant with risk factors, signs, or symptoms of infection presenting before 3 months of age had a blood culture and nasopharyngeal swab taken to determine the etiology of neonatal and young infant sepsis.

**Results:**

Among 4492 blood cultures, 460 were obtained from HIV-exposed infants. Nine infants (1.9%) had positive blood cultures. The most frequently isolated organisms were *Escherichia coli*, group B *Streptococcus*, and *Streptococcus viridans*, and these organisms demonstrated resistance to the common antibiotics (aminoglycosides, penicillins, and cephalosporins) used for management of suspected sepsis. A higher proportion of the exposed babies died vs HIV-unexposed (15.8 vs 11.2; *P* = .005). Nasopharyngeal swabs were collected from 114 infants, with 7.9% positive for at least one virus or bacterium.

**Conclusions:**

Future work is needed to investigate why mortality among HIV-exposed infants persists despite maternal antiretroviral treatment. Antimicrobial resistance is an increasing concern in this setting.

With the widespread rollout of maternal antiretroviral therapy (ART), the numbers of HIV-exposed uninfected (HEU) children are increasing globally [1]. In 2020, 15.4 million HEU children were born to women with HIV. This represents an extra 600 000 HIV-exposed but uninfected children since 2018 [2]. Notably, about 90% of these reside in Sub-Saharan Africa [1].

A number of studies have independently confirmed that, compared with HIV-unexposed uninfected (HUU) children, HEU children are at a higher risk of morbidity and mortality [3–5]. This, in part, is attributed to factors associated with maternal HIV disease progression that impair placental immunoglobulin G transfer efficiency, thereby increasing the risk of disease among HEU children [6]. Furthermore, immune dysregulation including chronic immune activation and high levels of pro-inflammatory cytokines like tumor necrosis factor–α among women HIV leads to infection susceptibility and may impair immune function among HEUs [7]. HEU morbidity has been found to be strongly related to maternal HIV disease severity, with the morbidity being persistent until maternal CD4 counts were >800 cells/mL [5]. There are limited data in relation to HIV antiviral prophylaxis in infancy and infant infection risk in HEU.

Sepsis is one of the leading causes of mortality and morbidity among HEUs in low- and middle-income countries (LMICs) [5, 8]. Very limited data are available regarding the organisms responsible for this heightened risk of infectious morbidity among HEU children. A few studies have been conducted in Europe and some in Southern Africa, the majority during the pre-ART era. These studies report increased risk of invasive pneumococcal disease (IPD) and mortality among HEU compared with HUU children [8, 9]. Some studies cite group B *Streptococcus* (GBS) and *Streptococcus pneumoniae* as the predominant pathogens [10, 11]. Early-onset infection was 10 times more frequent in HEU children in a study from Spain, consistent with studies performed in Southern Africa [10]. Where sepsis is recorded, there are also limited data on antimicrobial sensitivity and resistance patterns among HEU neonates [12].

We therefore sought to describe the etiology of culture-confirmed sepsis and antibiotic resistance patterns among HIV-exposed infants in a large cohort study of Ugandan infants.

This paper forms part of a supplement based on the PROGRESS study. The Progressing Group B Streptococcal Vaccines (PROGRESS) study aimed to describe the causes of infectious mortality and morbidity as well as the seroepidemiology of group B streptococcal infection—the major cause of neonatal sepsis worldwide—in Kampala, Uganda. Detailed information regarding the PROGRESS research protocol and results has been published separately [13].

## METHODS

### Study Design

This was a prospective birth cohort study with surveillance of neonatal and young infant (<90 days) disease. Any infant presenting with signs or risk factors of sepsis to either of the study hospitals had blood cultures and nasopharyngeal swabs to determine the etiology of neonatal and young infant sepsis.

### Study Setting

The study was based at 2 Ugandan sites: 1 maternity national referral hospital (Kawempe National Referral Hospital KNRH, Kampala, Uganda) and 1 general national referral hospital (Mulago National Referral Hospital, Kampala, Uganda). Kawempe Hospital is the largest national referral hospital for pregnancies in Kampala, Uganda's capital city, taking high-risk pregnancies from across the surrounding areas and all deliveries from the local community. There is a 100-bed neonatal unit admitting all infants weighing >1000 g as well as neonates with birth-related complications, sepsis, or congenital anomalies. More details of the study population are described in the PROGRESS protocol paper [13]. Participant recruitment sites for the studies that form part of this supplement are also detailed in a flowchart available in the supplementary material of another paper published in this issue [14]. Mulago National Referral Hospital is a large hospital in Kampala with an official pediatric bed capacity of 78 children, although it is regularly at 300% capacity.

### Study Population

This was a prospective observational cohort study of infants presenting with signs or risk factors for infection (sepsis, pneumonia, or meningitis) that aimed to describe the etiology of bacterial and viral admissions in young infants, antimicrobial resistance in those with bloodstream infections, and factors associated with in-hospital mortality. Eligible participants included neonates and young infants (<90 days) presenting with signs of infection (sepsis, meningitis, or pneumonia). Details of the signs and symptoms sought can be found in Supplementary Table 1. Any eligible infant aged 0–90 days presenting to either of the study hospitals' neonatal and pediatric units with signs of infection or at least 2 risk factors for sepsis had a blood culture, lumbar puncture where indicated, and nasopharyngeal swab (NPS) collected as part of their routine care before receipt of antibiotics where possible.

### Neonatal Blood Culture

Collection of blood and CSF cultures occurred between April 2019 and December 31, 2021. A flocked nasopharyngeal swab (Copan 484CE), lumbar puncture if appropriate, and 1–2 mL of blood for culture was collected, and clinical information was abstracted from the medical records. A smaller sample was collected from very low-birthweight babies, according to their birthweight (Supplementary Table 2). The volume of blood taken was documented on the sample request form, and blood culture bottles were weighed before and after sample to check volumes. Blood was inoculated into BD BACTEC Peds Plus bottles and then sent for culture at Makerere University Clinical Microbiology Laboratory (MUCML). MUCML has been accredited by the College of American Pathologists for microbiological testing since 2018. Transport was arranged twice daily from the study sites to the Clinical & Laboratory Standards Institute–accredited laboratory, located within the central Kampala region.

### Blood Culture Systems

All blood cultures were incubated in an automatic BACTEC machine (BACTEC 9050, 9120 [Becton Dickinson, Plymouth, UK] and FX40 [Becton Dickinson, Franklin Lakes, NJ]). After 48 hours of incubation, a preliminary result was issued if the blood had not flagged positive, and the final report was issued after 5 days of incubation.

### Nasopharyngeal Swabs

Nasopharyngeal swabs were collected before receipt of antibiotics where possible. In the case of stockouts of NPS swabs, blood culture was taken only. Full laboratory methods including blood culture processing, antimicrobial susceptibility testing, and NPS PCR were published in the first paper in this supplement [14].

### Case Definitions

HIV-exposed neonates were defined as neonates born to women whose HIV status was known to be positive while HIV-unexposed neonates were defined as neonates born to women whose HIV status was known as negative based on the national HIV testing algorithm [15].

Culture-positive cases were defined as a preliminary positive result issued after 48 hours of incubation and final report issued after 5 days of incubation. Gestational age in the newborn was defined according to last known menstrual period, symphysis fundal height, ultrasound scan, or the Ballard Maturational Assessment (Ballard Score) depending on availability.

### Data Management and Analysis

Relevant clinical information was extracted from the infant's hand-held or hospital notes onto the case report form (CRF) in Research Electronic Data Capture (REDCap) [11] hosted at Makerere University–Johns Hopkins University (MUJHU).

## RESULTS

**Figure 1.**
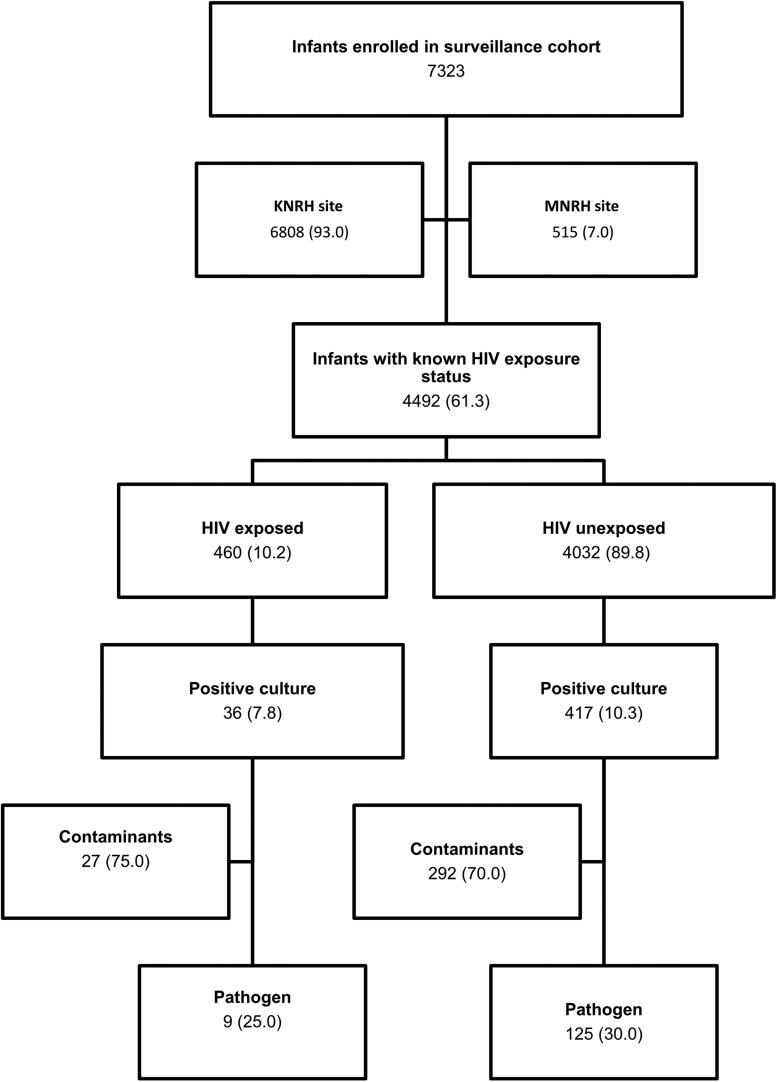
Flowchart showing culture results for HIV-exposed and -unexposed infants.

A total of 460 blood cultures were obtained from HIV-exposed infants (Figure 1 consort diagram). The majority of babies were delivered by spontaneous vaginal delivery (62.2%). The median birthweight for the HIV-exposed infants (interquartile range) was 2400 (1800–3100) g. A greater proportion of HIV-exposed infants were low birthweight compared with unexposed infants (50.7 vs 41.7; *P* = .003). A higher proportion of the exposed babies died during their admission compared with unexposed (15.8 vs 11.2; *P* = .003). Fewer HIV-exposed babies were admitted with suspected sepsis as the principal reason for admission compared with the HIV-unexposed babies (Table 1).

**Table 1.** Baseline Characteristics of HIV-Exposed and -Unexposed Infants

	HIV Exposure		
Variables	Unexposed	Exposed	*P* value
n = 4492	4032 (89.8)	460 (10.2)	…
Culture results (n = 4492)			
Pathogen negative	3907 (96.9)	451 (98.0)	.17
Pathogen positive	125 (3.1)	9 (2.0)	
Reason for admission (n = 4484)			
Sepsis/infection	1162 (28.9)	88 (19.2)	<.00001
Prematurity	1359 (33.8)	192 (41.8)	
Neonatal encephalopathy	1194 (29.7)	132 (28.8)	
Other	353 (8.8)	47 (10.2)	
Delivery mode (n = 4368)			
Spontaneous vaginal	2446 (62.3)	276 (62.2)	.37
C-section	1443 (36.8)	161 (36.3)	
Assisted vaginal delivery	35 (0.9)	7 (1.6)	
Baby gender (n = 4314)			
Male	2136 (55.2)	232 (52.3)	.24
Female	1734 (44.8)	212 (47.8)	
Birth location (n = 3902)			
Out-born (health facility)	968 (27.6)	121 (30.8)	.25
Out-born (home)	67 (1.9)	10 (2.5)	
In-born (study site)	2472 (70.5)	262 (66.7)	
Maturity at birth (n = 4088)			
Term	2203 (60.0)	215 (51.4)	.001
Preterm	1467 (40.0)	203 (48.6)	
Age at admission (n = 4366)			
0–6 d	3644 (93.0)	428 (95.5)	.04
≥7 d	274 (7.0)	20 (4.5)	
Birth weight (n = 2766)			
Median (IQR), g	2725 (1970–3300)	2400 (1800–3100)	…
<2500 g	1028 (41.7)	152 (50.7)	.003
≥2500 g	1438 (58.3)	148 (49.3)	
Outcome of admission (n = 4164)			
Died	417 (11.2)	67 (15.8)	.005
Alive	3322 (88.9)	358 (84.2)	
Signs of sepsis at admission (n = 4492)			
Risk factors only	393 (9.8)	46 (10.0)	.70
1–3 signs	2620 (65.0)	306 (66.5)	
>3 signs	1019 (25.3)	108 (23.5)	

Abbreviation: IQR, interquartile range.

### Confirmed Infections

Out of the 460 HIV-exposed infants who had a blood culture taken, 9 (2.0%) HIV exposed infants had positive blood cultures. 114 infants had an NPS swab collected, and 9 (7.9%) had a positive nasopharyngeal swab.

### Sensitivity Profile for the Isolated Organisms in Blood Cultures

The 9 organisms isolated among the HIV-exposed neonates included Group B *Streptococcus* (*S. agalactiae*; 33.3%), viridans *Streptococcus* (22.2%), *Escherichia coli* (22.1%), *Enterococcus* spp. (11.1%), and *Klebsiella pneumoniae* (11.1%) (Table 2).

**Table 2.** Pathogens Identified in HIV-Exposed Infants

Pathogens	Freq (%)
*Streptococcus agalactiae*	3 (33.3)
*Escherichia coli*	2 (22.2)
*Enterococcus faecium*	1 (11.1)
*Klebsiella pneumoniae*	1 (11.1)
*Streptococcus salivarius*	1 (11.1)
Viridans *Streptococcus*	1 (11.1)
Total	9 (100)

Three isolates were resistant to gentamicin (33.3%). In total, 5 isolates (55.6%) were resistant to penicillin (benzylpenicillin or ampicillin). Gram-negative isolates were tested for ceftazidime and ampicillin sensitivity; all isolates were sensitive to amikacin, and 66.7% were resistant to ceftazidime (Table 3).

**Table 3.** Antimicrobial Susceptibility of Pathogens Identified From HIV-Exposed Infants

Antimicrobial	Sensitive Freq (%)	Intermediate Freq (%)	Resistant Freq (%)	Total
Benzylpenicillin	3 (50)	1 (16.7)	2 (33.3)	6
Gentamicin	6 (66.7)	0 (0)	3 (33.3)	9
Ampicillin	0 (0)	0 (0)	3 (100)	3
Ceftazidime	1 (33.3)	0 (0)	2 (66.7)	3
Amikacin	3 (100)	0 (0)	0 (0)	3

### Nasopharyngeal Swab Results

Five infants had a viral organism isolated from their nasopharyngeal swab. The following viruses were identified: parainfluenza (n = 2), parainfluenza and influenza A (n = 1), influenza A and influenza B (n = 1), and rhinovirus (n = 1) (Table 4). Four bacterial organisms were identified in the NPS swabs of HIV-exposed infants: *Haemophilus influenzae* (n = 3), *Streptococcus pneumoniae* (n = 1) (Table 4).

**Table 4.** Nasopharyngeal Swab Results in HIV-Exposed Infants

NPS-Positive Results (n = 10)	Freq (%)
Rhinovirus	1 (11.1)
Influenza A & influenza B	1 (11.1)
Parainfluenza virus	2 (22.2)
Parainfluenza & influenza A	1 (11.1)
*Haemophilus influenzae*	3 (33.3)
*Streptococcus pneumoniae*	1 (11.1)
Total	9 (100)

## DISCUSSION

Our study demonstrates important information about neonatal sepsis in HIV-exposed infants in Uganda during the Test and Treat era. Despite early, optimal ART in the mothers of these infants, we have shown that HIV-exposed infants admitted with signs or risk factors of suspected sepsis have lower birthweight and higher case fatality compared with HIV-unexposed infants. This suggests an impact of maternal HIV infection on infant health, even in the absence of HIV infection in the infant. An association between HIV exposure and low birthweight and prematurity has been described previously. In a systematic review of 52 cohort studies, HIV infection was significantly associated with low birthweight, with a pooled odds ratio of 1.73 (95% CI, 1.64–1.82; *P* < .001), and preterm delivery, with an odds ratio of 1.56 (95% CI, 1.49–1.63; *P* < .001) [16]. We have reported elsewhere that after controlling for gestational age, age at admission, number of signs of sepsis, mode of delivery, and presence of a confirmed bloodstream infection, infants who are HIV-exposed have greater odds of dying during their inpatient admission than HIV-unexposed infants [14].

We found that the proportion of HIV-exposed neonates with pathogen-positive cultures was lower (2.0%) compared with HIV-unexposed infants (3.1%), although this difference was not statistically significant. This may be due to the universal provision of cotrimoxazole in our population during the first weeks of life, which may prevent culture-positive sepsis. The role of cotrimoxazole in prevention of infection in infants is still controversial, with a noninferiority trial in South Africa showing benefit and another trial in Malawi showing no benefit [17, 18].

*Streptococcus agalactiae* (GBS), *Streptococcus Viridans,* and *Enterococcus* species were isolated in two-thirds of the positive blood cultures among HIV-exposed infants. In the first paper in this series [14], we demonstrated that GBS sepsis occurred in 13.5% of all infants; however, when limited to HIV-exposed infants in this study, GBS accounted for a third of all infections. This is consistent with other studies from southern Africa that reported an higher risk of streptococcal infections in HIV-exposed uninfected infants than in the control population [8, 19].

Our study revealed high antimicrobial resistance to penicillin (55.6%) and aminoglycosides (33.3%) in organisms isolated in blood among HIV-exposed infants. The World Health Organization (WHO) Access group antibiotics (aminoglycosides and penicillin) are routinely used as first-line treatment for neonatal sepsis and should ideally have a low resistance potential. Third-generation cephalosporins like ceftazidime (33.3% resistance in our study) fall under the WHO Watch group and are high-priority agents and critically important antimicrobials for human medicine. This finding is worrying and could be contributing to high mortality rates among babies hospitalized with suspected sepsis.

Our study has several limitations; the small number of pathogen-positive cultures among HIV-exposed infants makes definitive comparisons of pathogens and resistance profiles between HIV-exposed and -unexposed infants difficult to make. We did not have information on maternal HIV status including clinical stage and viral load at delivery and whether the exposed babies developed HIV themselves.

In conclusion, the HIV-exposed infants in our study were more likely to die during their admission than HIV-unexposed infants. Streptococcal infections such as *Streptococcus agalactiae* seemed to be overrepresented, although the number of positive cultures was too small to draw definitive conclusions. They had high resistance to the commonly used first-line antibiotics. This has implications for effectively treating infections and reducing mortality in this important group.

## Supplementary Data

Supplementary materials are available at *Open Forum Infectious Diseases* online. Consisting of data provided by the authors to benefit the reader, the posted materials are not copyedited and are the sole responsibility of the authors, so questions or comments should be addressed to the corresponding author.

## Supplementary Material

ofae642_Supplementary_Data

## Notes

***Acknowledgments.*** We thank the women and children who participated in the PROGRESS study at each of the research sites. We would like to acknowledge the research teams and staff at MUJHU and Kawempe National Referral Hospital.

The complete PROGRESS Study Author Group is comprised of Abdelmajid Djennad, Agnes Nyamaizi, Agnes Ssali, Alexander Amone, Amusa Wamawobe, Annettee Nakimuli, Caitlin Farley, Carol Nanyunja, Christine Najuka, Cleophas Komugisha, Dan R. Shelley, Edward A. R. Portal, Ellie Duckworth, Emilie Karafillakis, Geraldine O’Hara, Godfrey Matovu, Hannah G. Davies, Janet Seeley, Joseph Peacock, Juliet Nsimire Sendagala, Katie Cowie, Kirsty Le Doare, Konstantinos Karampatsas, Lauren Hookham, Madeleine Cochet, Margaret Sewegaba, Mary Kyohere, Maxensia Owor, Melanie Etti, Merryn Voysey, Moses Musooko, Musa Sekikubo, Owen B. Spiller, Patience Atuhaire, Paul T. Heath, Philippa Musoke, Phiona Nalubega, Pooja Ravji, Richard Katungye, Ritah Namugumya, Rosalin Parks, Rose Azuba, Sam Kipyeko, Simon Beach, Stephen Bentley, Tim Old, Tobius Mutabazi, Valerie Tusubira, and Vicki Chalker.

***Author contributions*.** K.L.D., M.S., and P.M. conceived of the study design and methodology. M.K., H.G.D., and V.T. were responsible for the conduct of the study and oversight of data collection activities. Data management activities were managed by V.T. Laboratory analysis was overseen and undertaken by A.W. and J.N. K.L.D., P.A., H.G.D., V.T., and M.S. analyzed and interpreted the data. P.A., H.G.D., V.T., and J.P. drafted the manuscript, and all authors contributed to further revisions. All authors approved the final manuscript.

***Financial support*.** This work was supported by the Bill and Melinda Gates Foundation (OPP1189053). This work was also supported by the National Institute for Healthcare Research for the qualitative research (16/136/46) and by St George's, University of London (SGUL), R&D Protocol Code Number 17.0018.

***Disclaimer.*** The content of the manuscript is solely the responsibility of the authors. The funders had no role in study design, data collection and analysis, decision to publish, or preparation of the manuscript.

***Patient consent*.** Written informed consent was obtained from all patients involved in the study. The study has been approved by the Ugandan National Council for Science and Technology (UNCST; ref. HS 2496), Makerere University School of Medicine Research & Ethics Committee (SOMREC; 2018–130), and St George's, University of London (SGUL REC ref 2020.0024), which sponsored the study. The protocol was published in Gates Open Research in June 2020 (10).

***Supplement sponsorship***. This article appears as part of the supplement “Neonatal and Young Infant Infection in Uganda: the PROGRESS Study,” sponsored by St George's, University of London.

***Data availability*.** The full data set for the PROGRESS study is available at doi:10.24376/rd.sgul.24101115.v1 [20].
