# Supplementary material for: HIV Exposure and Neonatal Sepsis: A Descriptive Etiological Study
Source: Open Forum Infect Dis. 2025 Mar 10;11(Suppl 3):S187–92. doi: 10.1093/ofid/ofae642 (PMC11891132; doi:10.1093/ofid/ofae642)
Supplement: ofae642_Supplementary_Data [file ofae642_supplementary_data.docx]

Supplementary Table 1 - Clinical signs and symptoms

| Respiratory signs | chest indrawing, grunting, cyanosis, apnoea, oxygen requirement, |
| --- | --- |
| Neurological signs | seizures, hypotonia, irritability, lethargy, bulging fontanelle, no movement or movement only when stimulated. |
| Cardiovascular signs | delayed capillary refill time >3seconds, abnormal heartrate (>180 bpm or <100). |
| Gastrointestinal signs | difficulty feeding or feed intolerance, abdominal distension, diarrhoea, vomiting, signs of dehydration. |
| Skin signs | Jaundice, multiple or severe skin pustules, pus from the umbilical stump, petechial rash. |
| Other | Fever, hypothermia or temperature instability |

Supplementary Table 2 - Maximum appropriate blood volume to be drawn for study procedures according to weight of baby*

| **Weight of baby (kilograms)** | **Maximum volume of blood in single draw* (millilitres)** |
| --- | --- |
| **4** | 3.2 |
| **3** | 2.4 |
| **2** | 1.6 |
| **1.5** | 1.2 |
| **1.3** | 1.0 |
| **1.0** | 0.8 |
| **0.75** | 0.6 |
| **0.5** | 0.4 |

*Adapted from: ETHICAL CONSIDERATIONS FOR CLINICAL TRIALS ON MEDICINAL PRODUCTS CONDUCTED WITH THE PAEDIATRIC POPULATION Recommendations of the ad hoc group for the development of implementing guidelines for Directive 2001/20/EC relating to good clinical practice in the conduct of clinical trials on medicinal products for human use.

Supplementary Figure 1 - Flowchart showing recruitment sites for the studies included in this OFID supplement issue.

*Results of this birth cohort have been reported elsewhere (once accepted reference the PROGRESS main paper).

^#^ Includes HIV exposed and unexposed infants and infants form amongst the birth cohort.
